# Intimate Partner Violence against Women Living in Inadequate Conditions in Sub-Saharan Africa: A Meta-Analysis of Demographic and Health Survey Data

**DOI:** 10.3390/ijerph181910138

**Published:** 2021-09-27

**Authors:** Yaqing Gao, Yinping Wang, Xiaoyi Mi, Mo Zhou, Siyu Zou, Hong Zhou

**Affiliations:** Department of Maternal and Child Health, School of Public Health/National Health Commission Key Laboratory of Reproductive Health, Peking University, Beijing 100191, China; gaoyaqing@bjmu.edu.cn (Y.G.); wyping201406@bjmu.edu.cn (Y.W.); 2011210093@bjmu.edu.cn (X.M.); gaoss@pku.edu.cn (M.Z.); zousiyu@pku.edu.cn (S.Z.)

**Keywords:** inadequate living conditions, intimate partner violence, sub-Saharan Africa, meta-analysis

## Abstract

Intimate partner violence (IPV) against women is a major public health problem and is widespread in sub-Saharan Africa (SSA). However, little is known about its environmental determinants. This study aimed to investigate whether inadequate living conditions are associated with IPV victimization in women in SSA. We analyzed cross-sectional data for 102,714 women in 25 SSA countries obtained from the Demographic and Health Surveys Program. Logistic regression was used to estimate the country-specific effects of inadequate living conditions (housing with at least one of four characteristics of unimproved water, unimproved sanitation, insufficient space, and unfinished materials) on multiple forms of IPV. Random effects meta-analysis was used to combined the country-specific estimates. We found an association between inadequate living conditions and a higher likelihood of experiencing any (OR = 1.12, 95% CI 1.03 to 1.23, *p* = 0.012), sexual (OR = 1.18, 95% CI 1.05 to 1.34, *p* = 0.008), emotional (OR = 1.12, 95% CI 1.02 to 1.23, *p* = 0.023), and physical (OR = 1.15, 95% CI 1.03 to 1.28, *p* = 0.010) IPV. The associations were stronger for rural and less-educated women. These findings suggest that future research to establish a causal link between living conditions and IPV and to elucidate the underlying pathways is crucial to design IPV interventions in SSA.

## 1. Introduction

Intimate partner violence (IPV) is increasingly recognized as a significant social and public health problem. Globally, almost one-third (30%) of ever-partnered women have experienced physical and/or sexual IPV [1]. Most IPV research of risk factors and interventions focused on individual factors, such as a woman’s or partner’s wealth, education, and acceptance of violence and women’s decision-making autonomy [1,2]. However, only a little attention has been paid to environmental determinants of IPV, such as drought [3] and armed conflict [4].

Inadequate living conditions, commonly characterized by unimproved water and sanitation, insufficient living area, and non-durable construction, are universal markers of a disadvantaged home environment [5]. Studies have revealed that inadequate living conditions are directly associated with poor health outcomes, such as injuries, infectious diseases, and malnutrition [6]. Inadequate living conditions have also been associated with depression and stress [7], which in turn may contribute to the occurrence of violence [8]. Indeed, associations of inadequate living conditions and/or its characteristics with multiple forms of violence have been documented, such as non-partner violence [9,10,11], homicide [12], and child abuse [13]. Thus, inadequate living conditions might also be linked to IPV toward women. However, few studies have investigated the direct associations between inadequate living conditions and multiple forms of IPV toward women.

Living conditions are particularly poor in sub-Saharan Africa (SSA), because of the high rate of population growth and lack of housing [14]. In 2015, 47% (53 million) of the urban population and 82% (595 million) of the rural population lived in unimproved housing in SSA [5]. Houses are deemed unimproved when they have at least one of the four characteristics of unimproved water, unimproved sanitation, more than three people per bedroom, and being made of unfinished materials [5]. SSA also has a high incidence of IPV, reflecting the situation on the African continent, where 36.6% of ever-partnered women are affected by IPV [1]. The association between inadequate living conditions and different forms of IPV merits examination in a multi-country setting, such as SSA.

In the present study, we used data collected by the Demographic and Health Surveys (DHS) Program [15] from 25 countries in SSA to test the hypothesis that, for married or cohabiting women, inadequate living conditions are associated with a higher likelihood of experiencing emotional, physical, and/or sexual violence. We conducted stratified analyses by residence type (rural/urban) and education level (less than secondary/secondary or higher education) of the respondent, which could help design targeted screening and interventions for vulnerable groups.

## 2. Materials and Methods

### 2.1. Data Source

DHS are cross-sectional surveys with a focus on households in low- and middle-income countries (LMICs), and provide nationally representative data on demography, reproductive health, and child health [15]. We selected DHS conducted in SSA countries that (1) included information on the housing environment; (2) assessed IPV using the standard questionnaire in the domestic violence module; and (3) were publicly available before July 2021. For countries that had completed multiple rounds of the DHS, we selected the most recent survey data as they represent the latest situation of that country.

The surveys use a randomized multi-stage cluster-sampling design. In the first stage, clusters are selected with probability being proportional to cluster size, which is defined by the national population census. In the second stage, a predetermined number (approximately 25–30) of households are randomly sampled within each cluster. The target population is all women aged 15 to 49 years. Information on IPV is collected from one woman who is randomly selected among all the eligible women within each household. We restricted our sample to women who provided information on IPV and were currently married or cohabiting with a male partner.

### 2.2. Outcomes

The DHS contains the domestic violence module, which was developed according to the guideline set out by the World Health Organization (WHO) on the ethical collection of such sensitive information [16]. The IPV questionnaire in the module is modified from the Conflict Tactics Scales, which has good validity and reliability [17]. The questionnaire includes questions that ask women whether their current or most recent (if divorced, separated, or widowed) husband/partner ever perpetrated any of a number of behaviorally specific acts of violence and the frequency of perpetration in the 12 months preceding the interview.

The IPV questionnaire assesses sexual, emotional, and physical violence. Supplementary Text S1 presents the complete IPV questionnaire. If a woman reported experiencing at least one of the acts belonging to a form of IPV in the past 12 months, she is defined as having experienced a specific form of IPV. “Any IPV” was defined by as the experience of any form of IPV in the past 12 months [18]. We selected all three forms of IPV and “any IPV” as outcomes.

### 2.3. Exposures

Based on the definition of a slum household proposed by the United Nations [19], we defined inadequate living conditions as housing lacking any one of the following characteristics: durable construction, improved water, improved sanitation, and sufficient living space. The definition is consistent with previous studies that assessed the association of housing with child physical and mental health in LMICs [20,21,22].

We used the quality of building materials as a proxy for a house’s durability. If the material of more than one out of three components of its floor, roof, and wall could be classified as “unfinished” based on the DHS criteria (Supplementary Table S2), then the house’s building material was considered as unfinished [5,20]. We classified the household access to water and sanitation as ‘improved’ and ‘unimproved’ using the WHO and the United Nations definition (Supplementary Table S3) [23]. A house provides insufficient living space if more than three usual residents shared the room for sleeping [5].

### 2.4. A Priori Confounding Variables

The following covariates were included to control for the confounding bias: household wealth quintiles, residence type (rural/urban), age and education of the respondent, age of the respondent’s partner, respondent’s marital (married/cohabiting) and employment (employed/not employed) status, whether the age at the start of the first marriage or union of the respondent was less than 18 years (yes/no), and whether the partner consumes alcohol (yes/no).

Age was categorized into 15–19, 20–29, 30–39, and 40–49 years for the respondent and categorized into 15–19, 20–29, 30–39, 40–49, 50–59, and 60+ years for her partner. Education was categorized as “no education”, “primary education”, “secondary education”, and “higher education”, based on the highest education level achieved by the respondent. We defined cohabiting women as those currently living with unmarried partners, and employed women as those currently working, at the time of the interview. DHS generates wealth quintiles for all respondents based on their household ownership of a number of assets using principal component analysis [24].

### 2.5. Statistical Analysis

We conducted multivariate logistic regression to assess the association between inadequate living conditions and IPV in each country, adjusted for *a priori* confounding variables. The outcome of interest was each of the three forms of IPV and any IPV, and the exposures were one of the four inadequate living condition characteristics and inadequate living conditions as a combined variable. Then, for each of the four outcomes in each country, we included all four inadequate living condition characteristics in a single model and examined every characteristic’s coefficients, which allows interactions between these characteristics to be taken into account.

We used the I^2^ index to measure the consistency of the estimates across countries [25], and the DerSimonian and Laird method for meta-analyses with random effects to pool the estimates for all countries [26].

To reveal the heterogeneities in the effect of inadequate living conditions on IPV, we stratified our analyses by residence type (rural/urban) and education level (less than secondary/secondary or higher education) of the respondent, adjusted for all a priori confounding variables except for the stratifying variable. We then pooled the results using random effects meta-analyses.

The proportion of missing data is below 3% for all variables in all countries (Supplementary Table S4). Given the small proportion of missing data and a missing at random assumption, the estimation bias would be allowable if we ignored the missing data [27]. Therefore, only data from respondents without missing information on exposures, outcomes, and confounding variables were included in the analyses. We considered the complex sample design of DHS (stratification, clustering, and survey weights for the domestic violence module) when we estimated the standard errors.

The results were considered as statistically significant at a *p*-value lower than 0.05. We used R version 3.6.1 (R Foundation, Vienna, Austria) for all analyses.

## 3. Results

### 3.1. Study Population

Data were extracted from surveys in 25 countries dating from 2008 to 2020. Complete outcomes, covariates, and exposure data were available for 102,714 women (Table 1). The median of the country-specific mean age of the women was 31.7 years, and that of their partners was 39.0 years. More than half of the women had less than secondary education in 22 out of the 25 countries. The percentage of unemployed women ranged from 11.1% in Ghana to 67.2% in Ethiopia. The highest proportion of women who were married or in a union before the age of 18 years was found in Chad (76.4%), followed by Ethiopia (62.3%) and Mali (61.0%).

### 3.2. Characteristics of Living Conditions and Intimate Partner Violence

#### 3.2.1. Living Conditions

More than half of the women lived in inadequate living conditions in all countries, except for Gabon and Gambia. The median percentage of the women living in inadequate living conditions was 74.2%. In Ethiopia (94.5%), Chad (93.6%), and Democratic Republic of the Congo (DRC) (91.8%), the percentages were >90% (Table 2). The median percentage of the women living in housing with unfinished material, unimproved water, unimproved sanitation, and insufficient room was 36.6%, 28.4%, 49.5%, and 27.8%, respectively. Ethiopia, Chad, and Mozambique had the highest percentage of the women who lived in housing with unfinished material (84.8%, 77.4%, and 76.2%, respectively), as well as the highest percentage of women who did not have access to improved sanitation (86.6%, 84.9%, and 72.9%, respectively). More than half of the women lived in housing with unimproved water only in DRC (51.6%). Crowdedness was particularly severe in Ethiopia (61.6%), Chad (41.6%), and Angola (38.4%).

#### 3.2.2. Intimate Partner Violence

The median prevalence of any, emotional, physical, and sexual IPV toward the women was as follows: any 32.5% (ranging from 7.6% in Comoros to 50.9% in Sierra Leone), emotional 24.7% (ranging from 5.7% in Comoros to 39.2% in Sierra Leone), physical 18.1% (ranging from 3.9% in Comoros to 38.9% in Sierra Leone), and sexual 6.4% (ranging from 1.1% in Comoros to 19.8% in Burundi) (Table 3).

### 3.3. Association of Inadequate Living Conditions with Intimate Partner Violence

After adjusting for *a priori* confounding variables, we found inadequate living conditions to be independently associated with a higher likelihood of experiencing any (OR = 1.12, 95% CI 1.03 to 1.23, *p* = 0.012), sexual (OR = 1.18, 95% CI 1.05 to 1.34, *p* = 0.008), emotional (OR = 1.12, 95% CI 1.02 to 1.23, *p* = 0.023), and physical (OR = 1.15, 95% CI 1.03 to 1.28, *p* = 0.010) IPV in the past 12 months (Table 4).

Positive and significant associations between inadequate living conditions and IPV were demonstrated in six countries for any IPV, three countries for sexual IPV, four countries for emotional IPV, and four countries for physical IPV (Supplementary Tables S5–S8). In Uganda, inadequate living conditions were associated with all three forms of IPV and any IPV. In Nigeria, inadequate living conditions were protective for all outcomes, except for sexual IPV. In Mali, inadequate living conditions were protective for emotional and any IPV.

Similar associations were found between insufficient space and any (OR = 1.10, 95% CI 1.03 to 1.18, *p* = 0.004), sexual (OR = 1.12, 95% CI 1.01 to 1.23, *p* = 0.030), emotional (OR = 1.12, 95% CI 1.06 to 1.20, *p* < 0.001), and physical (OR = 1.11, 95% CI 1.03 to 1.19, *p* = 0.007) IPV. Unimproved sanitation was associated with a 10% increase in the odds of experiencing physical IPV (OR = 1.10, 95% CI 1.01 to 1.20, *p* = 0.024). Unimproved water was associated with a 9% increase in the odds of experiencing any (OR = 1.09, 95% CI 1.02 to 1.17, *p* = 0.012) and emotional (OR = 1.09, 95% CI 1.03 to 1.16, *p* = 0.005) IPV.

The association between a specific characteristic of inadequate living conditions and IPV persisted after the additional adjustment for the other three characteristics, and the estimates were almost identical to those in the analyses that only adjusted for a priori confounding variables (Supplementary Table S9).

The association between inadequate living conditions and IPV was stronger among rural women and women with lower education levels (Table 5). Inadequate living conditions were associated with an increase in the odds of experiencing emotional (OR = 1.13, 95% CI 1.01 to 1.27, *p* = 0.034), physical (OR = 1.12, 95% CI 1.01 to 1.24, *p* = 0.025), and any (OR = 1.13, 95% CI 1.02 to 1.25, *p* = 0.020) IPV in rural women, but not in urban women. Women with less than secondary education who lived in inadequate living conditions had a higher risk of experiencing sexual (OR = 1.20, 95% CI 1.02 to 1.41, *p* = 0.031), emotional (OR = 1.13, 95% CI 1.00 to 1.27, *p* = 0.043), and any (OR = 1.12, 95% CI 1.01 to 1.25, *p* = 0.026) IPV. Among women with secondary or higher education, inadequate living conditions were associated only with experiencing physical IPV (OR = 1.16, 95% CI 1.00 to 1.34, *p* = 0.047).

## 4. Discussion

According to our literature search, the present research is the first multi-country study to examine the association between inadequate living conditions and IPV. Overall, we found that inadequate living conditions were associated with 13% higher odds of any IPV, 19% higher odds of sexual IPV, 12% higher odds of emotional IPV, and 15% higher odds of physical IPV. We also found that insufficient space was individually associated with experiencing one or more forms of IPV. The magnitudes of associations of unimproved water and unimproved sanitation with IPV were weak, although some were statistically significant. There were no statistically significant associations between unfinished material and any form of IPV.

Our findings add to the body of literature documenting the associations of inadequate living conditions and their characteristics with IPV in LMICs. Evidence from DHS data in Nepal suggests that unimproved household water sources elevated women’s risk of experiencing physical and emotional IPV [28]. A study conducted in Brazil suggests that 20–59-year-old women living in households with more than two people per bedroom were more likely to report experiencing IPV compared with those living in less crowded housing [29].

However, contrary to our hypothesis, we found two countries, namely Mali and Nigeria, where inadequate living conditions were protective for at least one form of IPV. One potential explanation is that, in these two countries, partners’ attitudes toward IPV are an important factor for the occurrence of IPV [30,31], which could reverse or mute the relation between inadequate living conditions and IPV [32]. In Nigeria, the protective effect of women’s education levels could be reversed if the community’s social norms justify IPV [31]. In Mali, the cash transfer program has been reported to reduce household poverty, but offer limited effects on reducing IPV, as cash might allow men to enact authority [30]. Further research is needed to elucidate the heterogeneity in these findings. Specifically, additional evidence may reveal the mechanisms underlying the negative association between inadequate living conditions and IPV.

The association between living conditions and IPV might be driven by several potential mechanisms: (1) Inadequate living conditions may act as direct stressors. For example, household crowding might lead to stress and depression by making it difficult for women to escape negative interactions [7,33], which are associated with both IPV perpetration and victimization [8,34]. (2) Inadequate living conditions might be directly associated with poor physical health outcomes [6], leading to health-related economic losses, and even household food insecurity [35], which is well established as causing negative emotional well-being among household members [36,37,38]. (3) Inadequate living conditions might affect occupants’ social interactions, which have been recognized as protective factors associated with reduced IPV [39]. The home is commonly regarded as a place for the establishment and maintenance of social ties [40]. Individuals living in an unimproved home might also be reluctant to invite guests into their homes because of the stigma associated with poor housing environments [41], or socially withdraw from others to reduce excessive and unwanted social interactions, which is a prominent feature of a crowded home [42]. Individuals living in crowded homes are less likely to seek support from peers or offer support to peers in need [42].

We observed that rural women were more susceptible to the effects of inadequate living conditions on IPV compared with urban women. Research has reported higher rates of violence among rural couples than among urban couples in SSA [43], potentially owing to the fewer community resources, sparse distribution of law enforcement, and more accepting attitude toward IPV in rural areas [44]. Therefore, rural women are more vulnerable to IPV, which might be exacerbated by inadequate living conditions. We also noted that the association between inadequate living conditions and IPV remained statistically significant only in women with less than secondary education, who were more likely to experience IPV compared with women with secondary or higher education in SSA [43]. A lower education level might create an increased risk of IPV victimization through negative mental status, such as low self-esteem, stress, and depression [45], thereby affecting IPV through synergism with inadequate living conditions. In addition, educated people more often have a stable social support network [46], which might mitigate the erosion of social relationships by inadequate living conditions. Thus, policy makers should prioritize rural and less-educated women when gathering resources for housing interventions and IPV screening for families living in inadequate living conditions.

This study has several limitations. First, owing to the cross-sectional design of DHS, our analysis could not derive a causal relation between inadequate living conditions and IPV. Second, the psychological and social mediators that we hypothesized had not been assessed in the DHS; therefore, we could not conduct a mediation analysis. Third, other characteristics of inadequate living conditions such as toxins (e.g., lead, solvents), noise, and insufficient daylight might also result in behavioral disturbances (e.g., aggression and violence) [47]. However, sufficiently detailed measures of these characteristics are not included in the DHS data. Fourth, although the validity of the IPV questionnaire in DHS has been confirmed [18], women might underreport IPV for fear of victimization or stigmatization [48]. Non-differential misclassification generally biases the results to null [49]. Fifth, in some countries, the percentages of inadequate living conditions were high, even above 90%, which could reduce the precision of the estimates. We believe that a meta-analysis would compensate for this limitation by calculating the inverse variance-weighted average of the country-specific estimates. Sixth, our analysis only included married and cohabitating women aged 15–49 years in SSA countries; therefore, the generalization of our findings to other populations should be taken with caution, especially given the cultural differences in IPV disclosure and the subjective acceptability of crowdedness across regions and countries [48,50].

The strength of this study is that we investigated the associations of inadequate living conditions with IPV using multiple nationally representative datasets and analyzed the data in each country in a unified manner. Compared with a single-country study, our work included 25 countries across SSA, and could thus provide a more generalizable estimation. Further, instead of merging data from all countries and conducting a one-stage analysis, we used meta-analyses, which allowed the effect of covariates to vary across countries [51].

Both current WHO guidelines and the technical package for preventing and responding to IPV from the Centers for Disease Control and Prevention (CDC) have primarily focused on legal reform, advocacy of gender equity, and the promotion of the empowerment of women, giving little attention to the living environment [52,53]. Although the CDC’s technical package suggests creating a protective environment as a prevention strategy, it does not specify physical living conditions [53]. However, given the relatively small magnitude of the associations found in this study, we do not recommend that scarce resources be shifted away from IPV interventions that have already been shown to be effective in SSA, such as educational meetings or workshops addressing expectations about gender roles and behavior in communication and conflict resolution [54]. Instead, we suggest conducting additional research with a higher level of evidence to provide a better understanding of the effect of living conditions on IPV.

## 5. Conclusions

To the best of our knowledge, this study is the first in SSA to demonstrate the direct associations of inadequate living conditions, especially insufficient space, with three forms of IPV. Specifically, our findings highlighted that the association is most apparent in rural women and women with less than secondary education. Therefore, research with rigorous study designs and measurements of the exposures, outcomes, and mediators is needed to establish a causal link between living conditions and IPV to elucidate the pathways behind this link and design cost-effective IPV screenings and interventions in SSA.

## Figures and Tables

**Table 1:** Demographic characteristics of the study participants.

Country	Survey Year	Total Sample (*N*) ^a^	Mean Age (Years) ^b^	Partner’s Mean Age (Years) ^b^	Education (%) ^c^	Cohabiting (%)	Unemployed (%)	Married/in Union before Age 18 (%)	Alcohol (%) ^d^	Rural (%)
Angola	2015–2016	6578	30.9	37.8	67.4	79.6	24.9	42.2	40.9	36.4
Benin	2017–2018	4106	31.7	38.9	84.6	21.1	16.1	41.2	27.2	60.8
Burundi	2016–2017	6359	32.2	37.9	89.1	26.7	12.6	26.4	67.1	89.8
DRC	2013–2014	4952	30.7	37.5	61.3	26.5	23.5	51.0	49.6	68.2
Cameroon	2018	3852	31.0	40.8	60.3	22.1	30.3	47.5	45.2	52.5
Ethiopia	2016	4062	31.3	39.0	89.2	2.1	67.2	62.3	29.4	83.9
Gabon	2012	3355	32.3	40.7	32.2	63.6	45.8	31.5	60.4	12.9
Ghana	2008	1552	32.5	40.3	51.5	21.4	11.1	39.0	36.4	58.2
Gambia	2019–2020	1768	32.1	43.9	63.3	0.4	40.3	44.5	1.6	31.1
Kenya	2014	3701	31.7	38.5	64.9	8.7	30.0	34.1	33.6	61.3
Comoros	2012	2230	31.1	41.4	64.6	9.2	58.3	40.0	2.0	67.6
Liberia	2019–2020	1958	32.8	39.2	65.6	50.8	28.5	43.3	39.9	46.2
Mali	2018	3202	30.4	41.4	85.3	0.5	41.4	61.0	3.7	78.1
Malawi	2015–2016	4590	30.2	35.6	78.2	6.6	32.8	51.2	29.0	83.3
Mozambique	2011	4828	30.3	36.5	87.6	33.8	57.6	52.1	38.3	70.3
Nigeria	2018	8231	32.0	42.0	58.0	3.8	28.7	50.6	22.0	57.0
Namibia	2013	1111	33.5	38.8	28.6	43.6	48.8	16.6	56.1	42.3
Sierra Leone	2019	3785	32.4	42.4	74.2	6.3	18.7	44.8	17.2	64.2
Senegal	2019	1355	31.8	42.9	84.1	0.0	51.3	38.6	1.4	58.5
Chad	2014–2015	3128	30.0	40.2	89.1	9.4	52.9	76.4	31.0	81.2
Togo	2013–2014	4719	32.4	40.2	76.2	21.3	17.5	36.0	46.5	60.9
Tanzania	2015–2016	6251	31.7	38.7	84.7	27.8	20.8	42.2	31.0	68.7
Uganda	2016	6273	30.6	36.8	72.4	49.9	20.4	47.0	41.3	77.5
Zambia	2018	6016	31.6	37.6	60.7	0.9	48.2	46.5	35.8	60.0
Zimbabwe	2015	4752	31.5	38.1	31.7	4.6	55.8	36.7	38.3	65.8
Total ^e^	-	102,714	31.7	39.0	67.4	21.1	30.3	43.3	35.8	61.3

^a^ Number of women without missing information on exposures, outcomes, and confounding variables. ^b^ Mean or percentage of the confounding variables considering the complex sample design of DHS (stratification, clustering, and survey weights for the domestic violence module). ^c^ Percentage of women who had less than secondary education. ^d^ Percentage of women whose partners consume alcohol. ^e^ Median of the mean or the percentage of the confounding variables of all countries. DRC, Democratic Republic of the Congo.

**Table 2:** Characteristics of living conditions of the study participants.

Country	Total Sample (*N*) ^a^	Inadequate Living Conditions (%) ^b^	Unfinished Material (%)	Unimproved Water (%)	Unimproved Sanitation (%)	Insufficient Room (%)
Angola	6578	68.2	44.3	32.0	32.4	38.4
Benin	4106	81.5	27.9	29.9	68.5	32.9
Burundi	6359	74.4	45.5	17.2	49.5	19.1
DRC	4952	91.8	74.8	51.6	60.5	36.8
Cameroon	3852	66.2	37.1	27.3	43.4	27.2
Ethiopia	4062	94.5	84.8	37.0	86.6	61.6
Gabon	3355	47.9	1.1	7.3	33.4	22.5
Ghana	1552	63.3	20.1	14.0	34.4	35.1
Gambia	1768	48.5	7.1	7.2	32.9	18.3
Kenya	3701	80.3	61.2	29.6	51.0	38.1
Comoros	2230	77.0	14.6	9.9	63.2	26.3
Liberia	1958	74.5	36.5	16.6	54.2	29.2
Mali	3202	71.2	49.0	29.7	42.0	22.2
Malawi	4590	68.3	55.1	12.7	16.7	25.4
Mozambique	4828	88.5	76.2	49.4	72.9	27.8
Nigeria	8231	70.3	23.0	28.4	45.1	34.0
Namibia	1111	60.5	31.1	11.6	50.8	20.1
Sierra Leone	3785	71.7	36.6	35.8	49.5	18.8
Senegal	1355	50.8	16.5	15.0	25.2	26.6
Chad	3128	93.6	77.4	42.1	84.9	41.6
Togo	4719	77.5	19.4	35.6	61.3	33.9
Tanzania	6251	75.3	32.9	38.8	66.0	24.3
Uganda	6273	81.8	49.6	21.3	64.5	37.2
Zambia	6016	74.2	43.5	28.4	45.6	35.5
Zimbabwe	4752	55.8	23.3	22.5	35.4	22.8
Total ^c^	102,714	74.2	36.6	28.4	49.5	27.8

^a^ Number of women without missing information on exposures, outcomes, and confounding variables. ^b^ Percentage of the inadequate living conditions and its characteristics. ^c^ Median of the percentage of the inadequate living conditions and its characteristics of all countries. DRC, Democratic Republic of the Congo.

**Table 3:** Percentage of the study participants experiencing intimate partner violence over the past 12 months in each country included in the analysis.

Country	Total Sample (*N*) ^a^	Any (%) ^b^	Emotional (%) ^c^	Physical (%) ^d^	Sexual (%) ^e^
Angola	6578	33.7	23.9	23.9	6.2
Benin	4106	32.5	29.5	11.0	5.6
Burundi	6359	33.8	17.2	18.9	19.8
DRC	4952	43.3	28.8	29.8	18.8
Cameroon	3852	31.2	22.4	19.3	6.1
Ethiopia	4062	26.9	20.2	17.1	7.6
Gabon	3355	40.4	27.0	29.1	11.2
Ghana	1552	34.2	29.8	16.9	4.6
Gambia	1768	17.8	14.3	8.8	2.2
Kenya	3701	33.1	23.8	22.2	9.4
Comoros	2230	7.6	5.7	3.9	1.1
Liberia	1958	47.0	35.7	35.0	7.2
Mali	3202	34.4	28.4	18.1	7.7
Malawi	4590	32.3	22.5	15.4	14.8
Mozambique	4828	40.2	30.0	25.7	6.4
Nigeria	8231	29.7	27.0	11.4	4.3
Namibia	1111	26.9	20.4	17.8	5.9
Sierra Leone	3785	50.9	39.2	38.9	5.8
Senegal	1355	9.3	6.1	4.7	3.0
Chad	3128	22.9	15.9	15.3	6.2
Togo	4719	28.0	25.1	10.5	4.8
Tanzania	6251	38.1	27.9	26.9	9.0
Uganda	6273	41.1	30.7	22.7	16.1
Zambia	6016	32.3	22.3	20.8	10.5
Zimbabwe	4752	31.2	24.7	15.7	7.4
Total ^e^	102,714	32.5	24.7	18.1	6.4

^a^ Number of women without missing information on exposures, outcomes, and confounding variables. ^b^ Estimates of the percentage of the women who had experienced emotional IPV in the previous 12 months. ^c^ Estimates of the percentage of the women who had experienced physical IPV in the previous 12 months. ^d^ Estimates of the percentage of the women who had experienced sexual IPV in the previous 12 months. ^e^ Estimates of the median of the percentage of the characteristics of all countries. DRC, Democratic Republic of the Congo.

**Table 4:** Associations of housing characteristics with intimate partner violence.

Associations	OR (95% CI)	*p*-Value	I^2^ (%)
Unfinished material			
Sexual IPV	1.14 (0.99 to 1.32)	0.070	51.7
Emotional IPV	1.04 (0.96 to 1.14)	0.323	38.2
Physical IPV	1.06 (0.95 to 1.17)	0.287	47.4
Any IPV	1.07 (0.99 to 1.16)	0.099	42.3
Unimproved water			
Sexual IPV	1.07 (0.97 to 1.18)	0.164	34.8
Emotional IPV	1.09 (1.03 to 1.16)	0.005	23.9
Physical IPV	1.06 (0.98 to 1.15)	0.157	48.4
Any IPV	1.09 (1.02 to 1.17)	0.012	45.0
Unimproved sanitation			
Sexual IPV	1.02 (0.91 to 1.14)	0.734	43.2
Emotional IPV	1.07 (0.97 to 1.17)	0.159	59.7
Physical IPV	1.10 (1.01 to 1.20)	0.024	51.6
Any IPV	1.08 (1.00 to 1.17)	0.063	57.6
Insufficient space			
Sexual IPV	1.12 (1.01 to 1.23)	0.030	47.0
Emotional IPV	1.12 (1.06 to 1.20)	<0.001	44.8
Physical IPV	1.11 (1.03 to 1.19)	0.007	50.8
Any IPV	1.10 (1.03 to 1.18)	0.004	57.6
Inadequate living conditions			
Sexual IPV	1.18 (1.05 to 1.34)	0.008	30.7
Emotional IPV	1.12 (1.02 to 1.23)	0.023	52.8
Physical IPV	1.15 (1.03 to 1.28)	0.010	53.2
Any IPV	1.12 (1.03 to 1.23)	0.012	56.4

OR, odds ratio; CI, confidence interval; IPV, intimate partner violence.

**Table 5:** Stratified associations of inadequate living conditions with intimate partner violence.

Associations	OR (95% CI)	*p*-Value	I^2^ (%)
Rural			
Sexual IPV	1.19 (1.00 to 1.43)	0.051	28.5
Emotional IPV	1.13 (1.01 to 1.27)	0.034	31.0
Physical IPV	1.12 (1.01 to 1.24)	0.025	0.0
Any IPV	1.13 (1.02 to 1.25)	0.020	24.5
Urban			
Sexual IPV	1.11 (0.94 to 1.32)	0.212	32.4
Emotional IPV	1.10 (0.97 to 1.24)	0.133	50.1
Physical IPV	1.13 (0.98 to 1.31)	0.086	55.7
Any IPV	1.11 (0.97 to 1.26)	0.125	60.4
Less than secondary education			
Sexual IPV	1.20 (1.02 to 1.41)	0.031	30.8
Emotional IPV	1.13 (1.00 to 1.27)	0.043	44.3
Physical IPV	1.13 (1.00 to 1.27)	0.052	35.7
Any IPV	1.12 (1.01 to 1.25)	0.026	40.4
Secondary or higher education			
Sexual IPV	1.12 (0.97 to 1.30)	0.120	0.0
Emotional IPV	1.09 (0.97 to 1.22)	0.156	26.7
Physical IPV	1.16 (1.00 to 1.34)	0.047	39.6
Any IPV	1.11 (0.99 to 1.24)	0.076	33.2

OR, odds ratio; CI, confidence interval; IPV, intimate partner violence.

## Data Availability

The data presented in this study are openly available at https://dhsprogram.com/data (accessed on 31 July 2021). Registration is required for access to data.

## Supplementary Materials

The following are available online at https://www.mdpi.com/article/10.3390/ijerph181910138/s1, Table S1: The intimate partner violence (IPV) questionnaire in the Demographic and Health Surveys. Table S2: The Demographic and Health Surveys Program classifications of finished/unfinished materials. Table S3: The World Health Organization and United Nations Children’s Fund Joint Monitoring Programme for Water Supply, Sanitation, and Hygiene classifications of improved/unimproved facility types. Table S4: Percentage of participants with missing data for each variable of interest in each country. Table S5: Association of inadequate living conditions with any intimate partner violence in 25 countries. Table S6: Association of inadequate living conditions with sexual intimate partner violence in 25 countries. Table S7: Association of inadequate living conditions with emotional intimate partner violence in 25 countries. Table S8: Association of inadequate living conditions with physical intimate partner violence in 25 countries. Table S9: Mutually adjusted associations of inadequate living conditions and its characteristics with intimate partner violence.
