# Supplementary material for: Intimate Partner Violence against Women Living in Inadequate Conditions in Sub-Saharan Africa: A Meta-Analysis of Demographic and Health Survey Data
Source: Int J Environ Res Public Health. 2021 Sep 27;18(19):10138. doi: 10.3390/ijerph181910138 (PMC8507939; doi:10.3390/ijerph181910138)
Supplement: Supplementary file 1 [file ijerph-18-10138-s001.zip › ijerph-1340209-supplementary.pdf]

Text S1. The intimate partner violence (IPV) questionnaire in the Demographic and Health Surveys.

Now if you will permit me, I need to ask some more questions about your relationship with your (last) husband/partner.

(Does/did) your (last) husband/partner ever…during the last 12 months?

1. (emotional IPV) say or do something to humiliate you in front of others?
2. (emotional IPV) threaten to hurt or harm you or someone you care about?
3. (emotional IPV) insult you or make you feel bad about yourself?
4. (physical IPV) push you, shake you, or throw something at you?
5. (physical IPV) slap you?
6. (physical IPV) twist your arm or pull your hair?
7. (physical IPV) punch you with his fist or with something that could hurt you?
8. (physical IPV) kick you, drag you, or beat you up?
9. (physical IPV) try to choke you or burn you on purpose?
10. (physical IPV) threaten you with a knife, gun, or other weapon?
11. (sexual IPV) physically force you to have sexual intercourse with him when you did not want to?
12. (sexual IPV) physically force you to perform any other sexual acts you did not want to?

Table S2. The Demographic and Health Surveys Program classifications of finished/unfinished materials.

	**Wall**	**Roof**	**Floor**
Finished	Cement<br>Stone with lime / cement<br>Bricks<br>Cement blocks<br>Covered adobe<br>Wood planks / shingles<br>Burnt bricks<br>Metal<br>Bamboo with cement<br>Asbestos	Metal<br>Wood<br>Calamine / Cement fiber<br>Ceramic tiles<br>Cement<br>Roofing shingles<br>Clay tiles<br>Asbestos	Parquet or polished wood<br>Vinyl or asphalt strips<br>Ceramic tiles<br>Cement<br>Carpet<br>Terrazzo
Unfinished	No Walls<br>Cane / Palm / Trunks<br>Dirt<br>Bamboo with mud<br>Stone with mud<br>Uncovered adobe<br>Plywood<br>Cardboard<br>Reused wood<br>Bamboo mat<br>Bamboo/bamboo with dry leaf<br>Bamboo lattice<br>Unburnt bricks<br>Straw<br>Mud blocks	No Roof<br>Thatch / Palm leaf<br>Sod<br>Rustic mat<br>Palm / Bamboo<br>Wood planks<br>Cardboard<br>Canvas<br>Earth<br>Mud<br>Tarpaulin / plastic	Earth / Sand<br>Dung<br>Wood planks<br>Palm / Bamboo<br>Stones<br>Matting

Table S3. The World Health Organization and United Nations Children's Fund Joint Monitoring Programme for Water Supply, Sanitation and Hygiene classifications of improved/unimproved facility types.

	**Drinking water**	**Sanitation**
Improved	Tap water in the dwelling, yard or plot<br>Public standposts<br>Boreholes/tubewells<br>Protected wells and springs<br>Rainwater<br>Packaged water, including bottled water and sachet water<br>Delivered water, including tanker trucks and small carts	Flush and pour flush toilets connected to sewers<br>Flush and pour flush toilets or latrines connected to septic tanks or pits<br>Ventilated improved pit latrines<br>Pit latrines with slabs<br>Composting toilets, including twin pit latrines and container-based systems
Unimproved	Unprotected wells and springs<br>Surface water	Pit latrines without slabs<br>Hanging latrines<br>Bucket latrines<br>Open defecation

Table S4. Percentage of participants with missing data for each variable of interest in each country.

Country	Total (N) [a]	Sample Size (N) [b]	Mean Age [c]	Partner's Mean Age	Education	Cohabiting	Unemployed	Married/in union Before Age 18	Alcohol	Rural	Emotional IPV	Physical IPV	Sexual IPV
Angola	6578	6578	0.0	0.0	0.0	0.0	0.0	0.0	0.0	0.0	0.0	0.0	0.0
Benin	4106	4106	0.0	0.0	0.0	0.0	0.0	0.0	0.0	0.0	0.0	0.0	0.0
Burundi	6359	6359	0.0	0.0	0.0	0.0	0.0	0.0	0.0	0.0	0.0	0.0	0.0
DR Congo	5038	4952	0.0	0.8	0.0	0.0	0.2	0.0	0.0	0.0	0.2	0.4	0.3
Cameroon	3852	3852	0.0	0.0	0.0	0.0	0.0	0.0	0.0	0.0	0.0	0.0	0.0
Ethiopia	4062	4062	0.0	0.0	0.0	0.0	0.0	0.0	0.0	0.0	0.0	0.0	0.0
Gabon	3460	3355	0.0	0.0	0.0	0.0	0.2	0.0	0.7	0.0	1.2	2.2	1.6
Ghana	1570	1552	0.0	0.0	0.0	0.0	0.4	0.0	0.1	0.0	0.2	0.3	0.1
Gambia	1768	1768	0.0	0.0	0.0	0.0	0.0	0.0	0.0	0.0	0.0	0.0	0.0
Kenya	3770	3701	0.0	0.8	0.0	0.0	0.0	0.0	0.2	0.0	0.7	0.7	0.4
Comoros	2270	2230	0.0	0.0	0.3	0.0	0.3	0.0	0.1	0.0	0.3	0.5	0.0
Liberia	1958	1958	0.0	0.0	0.0	0.0	0.0	0.0	0.0	0.0	0.0	0.0	0.0
Mali	3202	3202	0.0	0.0	0.0	0.0	0.0	0.0	0.0	0.0	0.0	0.0	0.0
Malawi	4590	4590	0.0	0.0	0.0	0.0	0.0	0.0	0.0	0.0	0.0	0.0	0.0
Mozambique	4828	4828	0.0	0.0	0.0	0.0	0.0	0.0	0.0	0.0	0.0	0.0	0.0
Nigeria	8231	8231	0.0	0.0	0.0	0.0	0.0	0.0	0.0	0.0	0.0	0.0	0.0
Namibia	1234	1111	0.0	2.1	0.0	0.0	0.3	0.0	0.0	0.0	0.5	0.4	0.2
Sierra Leone	3785	3785	0.0	0.0	0.0	0.0	0.0	0.0	0.0	0.0	0.0	0.0	0.0
Senegal	1355	1355	0.0	0.0	0.0	0.0	0.0	0.0	0.0	0.0	0.0	0.0	0.0
Chad	3310	3128	0.0	2.7	0.0	0.0	1.3	0.0	0.2	0.0	1.0	0.7	0.7
Togo	4773	4719	0.0	0.3	0.0	0.0	0.1	0.0	0.1	0.0	0.4	0.3	0.1
Tanzania	6252	6251	0.0	0.0003	0.0	0.0	0.0	0.0	0.0	0.0	0.0	0.0	0.0
Uganda	6273	6273	0.0	0.0	0.0	0.0	0.0	0.0	0.0	0.0	0.0	0.0	0.0
Zambia	6016	6016	0.0	0.0	0.0	0.0	0.0	0.0	0.0	0.0	0.0	0.0	0.0
Zimbabwe	4754	4752	0.0	0.0003	0.0	0.0	0.0	0.0	0.0	0.0	0.0	0.0	0.0

[a] Number of women who were selected for the domestic violence module, were currently married or cohabiting with a male partner, and provided information on exposures (housing environments). [b] Number of women without missing information on housing environments, IPV, and confounding variables. [c] Percentage of participants with missing data for the variable of interest, considering the sample weight and the cluster and sample strata statements, based on the country-specific study design.

Table S5. Association of inadequate living conditions with any intimate partner violence in 25 countries.

Country	OR (95% CI)	*p*-Value
Angola	0.92 (0.70 to 1.22)	0.574
Benin	1.17 (0.89 to 1.52)	0.259
Burundi	1.04 (0.88 to 1.23)	0.633
DR Congo	1.51 (0.96 to 2.36)	0.073
Cameroon	0.92 (0.71 to 1.18)	0.505
Ethiopia	1.29 (0.73 to 2.27)	0.383
Gabon	0.83 (0.62 to 1.11)	0.211
Ghana	1.10 (0.81 to 1.49)	0.553
Gambia	1.64 (1.11 to 2.43)	0.012
Kenya	1.48 (1.02 to 2.14)	0.037
Comoros	1.00 (0.63 to 1.60)	0.995
Liberia	1.09 (0.71 to 1.69)	0.693
Mali	0.74 (0.57 to 0.97)	0.031
Malawi	1.25 (0.99 to 1.58)	0.066
Mozambique	1.45 (1.06 to 1.98)	0.021
Nigeria	0.79 (0.65 to 0.97)	0.022
Namibia	1.29 (0.68 to 2.45)	0.442
Sierra Leone	1.07 (0.80 to 1.43)	0.648
Senegal	1.84 (1.03 to 3.30)	0.040
Chad	1.10 (0.56 to 2.17)	0.776
Togo	0.98 (0.78 to 1.23)	0.878
Tanzania	1.44 (1.14 to 1.82)	0.002
Uganda	1.48 (1.17 to 1.88)	0.001
Zambia	1.09 (0.83 to 1.43)	0.524
Zimbabwe	1.18 (0.96 to 1.46)	0.118

OR, odds ratio; CI, confidence interval.

Table S6. Association of inadequate living conditions with sexual intimate partner violence in 25 countries.

Country	OR (95% CI)	*p* Value
Angola	1.16 (0.69 to 1.96)	0.577
Benin	1.12 (0.67 to 1.88)	0.666
Burundi	1.04 (0.85 to 1.27)	0.690
DR Congo	1.21 (0.69 to 2.11)	0.508
Cameroon	1.42 (0.91 to 2.21)	0.122
Ethiopia	1.06 (0.25 to 4.55)	0.934
Gabon	1.09 (0.66 to 1.79)	0.732
Ghana	1.32 (0.66 to 2.61)	0.430
Gambia	2.44 (0.96 to 6.15)	0.060
Kenya	1.65 (0.90 to 3.03)	0.108
Comoros	1.47 (0.39 to 5.62)	0.570
Liberia	0.51 (0.30 to 0.88)	0.016
Mali	0.93 (0.50 to 1.73)	0.815
Malawi	1.49 (1.10 to 2.02)	0.011
Mozambique	1.41 (0.76 to 2.63)	0.278
Nigeria	0.80 (0.55 to 1.16)	0.237
Namibia	3.85 (1.07 to 13.85)	0.039
Sierra Leone	1.59 (0.79 to 3.17)	0.190
Senegal	2.35 (0.69 to 8.07)	0.174
Chad	0.84 (0.14 to 4.94)	0.843
Togo	1.68 (0.90 to 3.13)	0.100
Tanzania	1.26 (0.87 to 1.82)	0.227
Uganda	1.50 (1.07 to 2.09)	0.019
Zambia	1.07 (0.76 to 1.50)	0.691
Zimbabwe	0.83 (0.57 to 1.21)	0.330

OR, odds ratio; CI, confidence interval.

Table S7. Association of inadequate living conditions with emotional intimate partner violence in 25 countries.

Country	OR (95% CI)	*p* Value
Angola	0.92 (0.67 to 1.26)	0.599
Benin	1.20 (0.90 to 1.58)	0.211
Burundi	1.13 (0.90 to 1.41)	0.309
DR Congo	1.40 (0.84 to 2.34)	0.202
Cameroon	1.10 (0.86 to 1.42)	0.436
Ethiopia	1.25 (0.65 to 2.38)	0.502
Gabon	0.88 (0.63 to 1.23)	0.449
Ghana	1.02 (0.73 to 1.43)	0.889
Gambia	1.41 (0.88 to 2.27)	0.153
Kenya	1.52 (1.09 to 2.13)	0.015
Comoros	1.19 (0.67 to 2.11)	0.547
Liberia	1.09 (0.66 to 1.81)	0.741
Mali	0.70 (0.54 to 0.91)	0.007
Malawi	1.17 (0.90 to 1.51)	0.252
Mozambique	1.28 (0.94 to 1.74)	0.112
Nigeria	0.79 (0.64 to 0.97)	0.024
Namibia	1.20 (0.63 to 2.25)	0.580
Sierra Leone	1.03 (0.78 to 1.35)	0.840
Senegal	1.77 (0.93 to 3.39)	0.083
Chad	1.02 (0.43 to 2.38)	0.969
Togo	0.89 (0.70 to 1.14)	0.359
Tanzania	1.49 (1.13 to 1.96)	0.004
Uganda	1.49 (1.16 to 1.92)	0.002
Zambia	1.03 (0.77 to 1.37)	0.863
Zimbabwe	1.33 (1.08 to 1.65)	0.009

OR, odds ratio; CI, confidence interval.

Table S8. Association of inadequate living conditions with physical intimate partner violence in 25 countries.

Country	OR (95% CI)	*p* Value
Angola	1.01 (0.78 to 1.31)	0.951
Benin	1.90 (1.22 to 2.96)	0.004
Burundi	1.02 (0.85 to 1.24)	0.815
DR Congo	1.12 (0.68 to 1.86)	0.649
Cameroon	0.92 (0.68 to 1.24)	0.589
Ethiopia	1.42 (0.70 to 2.91)	0.331
Gabon	0.82 (0.60 to 1.12)	0.210
Ghana	1.26 (0.86 to 1.86)	0.237
Gambia	2.40 (1.50 to 3.84)	0.000
Kenya	1.54 (0.93 to 2.57)	0.094
Comoros	0.87 (0.45 to 1.68)	0.672
Liberia	0.98 (0.63 to 1.51)	0.912
Mali	0.91 (0.65 to 1.28)	0.596
Malawi	1.16 (0.83 to 1.61)	0.390
Mozambique	1.61 (1.10 to 2.36)	0.013
Nigeria	0.79 (0.63 to 0.99)	0.040
Namibia	1.63 (0.89 to 2.98)	0.110
Sierra Leone	1.02 (0.78 to 1.35)	0.872
Senegal	2.12 (0.85 to 5.32)	0.109
Chad	0.60 (0.27 to 1.32)	0.205
Togo	1.15 (0.80 to 1.65)	0.457
Tanzania	1.15 (0.86 to 1.53)	0.337
Uganda	1.61 (1.16 to 2.23)	0.004
Zambia	1.15 (0.84 to 1.57)	0.382
Zimbabwe	1.22 (0.94 to 1.58)	0.138

OR, odds ratio; CI, confidence interval.

Table S9. Mutually adjusted associations of inadequate living conditions and its characteristics with intimate partner violence (adjusted for all confounding variables and the other three characteristics of inadequate living conditions).

	OR (95% CI)	*p* Value	I^2 (%)
Unfinished material			
Sexual IPV	1.13 (0.98 to 1.29)	0.086	47.2
Emotional IPV	1.04 (0.96 to 1.14)	0.335	37.6
Physical IPV	1.06 (0.96 to 1.18)	0.262	49.9
Any IPV	1.07 (0.99 to 1.16)	0.104	43.5
Unimproved water			
Sexual IPV	1.07 (0.97 to 1.19)	0.161	38.4
Emotional IPV	1.09 (1.02 to 1.16)	0.008	25.0
Physical IPV	1.05 (0.97 to 1.14)	0.226	48.7
Any IPV	1.09 (1.01 to 1.17)	0.021	47.7
Unimproved sanitation			
Sexual IPV	1.01 (0.91 to 1.13)	0.824	41.0
Emotional IPV	1.06 (0.97 to 1.16)	0.193	58.8
Physical IPV	1.10 (1.01 to 1.20)	0.026	51.8
Any IPV	1.07 (0.99 to 1.16)	0.088	57.2
Insufficient space			
Sexual IPV	1.12 (1.01 to 1.23)	0.027	45.6
Emotional IPV	1.13 (1.06 to 1.20)	<0.001	42.9
Physical IPV	1.11 (1.03 to 1.20)	0.006	52.2
Any IPV	1.11 (1.03 to 1.18)	0.003	57.9

OR, odds ratio; CI, confidence interval; IPV, intimate partner violence.
